# Special Issue: Therapeutic Potential for Cannabis and Cannabinoids

**DOI:** 10.3390/biomedicines11030902

**Published:** 2023-03-14

**Authors:** Wesley M. Raup-Konsavage

**Affiliations:** Department of Pharmacology, Penn State College of Medicine, Hershey, PA 17033, USA; wkonsavage@pennstatehealth.psu.edu; Tel.: +717-531-4172

The number of patients reporting the use of cannabis for medical purposes, whether through state-regulated medical marijuana programs or through over-the-counter hemp extracts, continues to grow. The growth in medicinal use of cannabis has in many ways surpassed the scientific data on the benefits and hazards of cannabis, and the scientific community has largely been left playing catch-up. Since 1996, when California became the first jurisdiction to legalize medical cannabis, the number of states following suit has grown and is currently at 37, while nearly 50 countries have legalized medical cannabis (and even more have decriminalized the plant) including Canada, Austria, Uruguay, Australia, South Korea, and Lesotho.

*Cannabis* sp. produces a number of phytochemicals with potential medical benefits including terpenes, flavonoids, and a unique class of molecules called cannabinoids, of which Δ^9^-tetrahydrocannabinol (THC) and cannabidiol (CBD) are the two most studied [1,2]. Amazingly, the plant produces over 100 different cannabinoids with different potential therapeutic targets and activities, and these remain understudied. The therapeutic benefits of cannabinoids are due, in large part, to the endocannabinoid system that exists in the human body, in addition to the ability of cannabinoids to interact and signal through a large number of disparate receptor molecules [3].

The recent growth in cannabis and cannabinoid research is perhaps best highlighted by the establishment of three scholarly journals devoted solely to this topic in the past few years, namely, *Cannabis and Cannabinoid Research*, in 2016, *Medical Cannabis and Cannabinoids*, in 2018, and *The Journal of Cannabis*, in 2019.

## 1. Original Research Articles

This Special Issue features fourteen original research articles across a wide range of topics and includes 10 reports on phytocannabinoids, 2 studies examining the endocannabinoid system, and 2 papers using synthetic cannabinoids. Three of these studies looked at the role of cannabinoids at mediating pain, two studies examined cannabinoids for treating mental illness, two studies addressed the potential safety of cannabinoids, two looked at cannabinoids as treatment options for gastrointestinal inflammation, and two studies examined the impact of cannabinoids on neurodevelopmental diseases. Other studies examined the impact of cannabinoids on cancer cell growth, anti-inflammatory activity in fibroblasts from patients with rheumatoid arthritis, regulation of matrix metalloproteases and cell proliferation, and regulation of neuroprotective genes in the brain. These various topics highlight the wide-ranging potential benefit of cannabis and cannabinoids to treat an array of human illness and disease.

In a study by Moreno-Sanz and colleagues, the impact of inhaled cannabis to treat pain and anxiety was examined [4]. The authors found that inhalation of pharmaceutical-grade cannabis flower provides patient-reported improvements to pain, mood, anxiety, and sleep. Perhaps most importantly the study reports an overall increased quality of life in a treatment-resistant group of patients. Sepulveda et al. found that pure THC, unlike pure CBD, is capable of reducing hyperalgesia in a murine model of chemotherapeutic-induced peripheral neuropathy [5]. This study also provides support for an “entourage” effect because a high CBD hemp extract, unlike the pure CBD, was able to reduce hyperalgesia when normalized to CBD levels; however, high THC extract and pure THC were found to offer the maximum reduction in sensitivity. In a study by Trevino and collaborators, the authors report that levels of the endocannabinoid 2-arachidonoylglcerol (2-AG) in the plasma at time of injury are positively correlated with chronic pain [6]. This suggests that increased activation of the endocannabinoid system can contribute to the development of chronic pain following an injury.

In addition to the Moreno-Sanz study described above, one other study examined cannabinoids in mental illness. A study by Zer-Aviv et al., found that inhibition of the endocannabinoid metabolizing enzyme fatty acid amide hydrolase (FAAH) produces a stress-protective effect through activation of β-catenin in the nucleus accumbens region of the brain [7]. The study also found that the increase in anandamide levels, from inhibition of FAAH, acts through the cannabinoid receptor 1 to increase nuclear levels of β-catenin.

Hajjar and colleagues examined the use of prescription and non-prescription medications in patients using medical cannabis, as self-reported by patients [8]. The study found that patients frequently switch medical cannabis products, which may be due to the need to find a dose and product that work well for the patient. The study also found that despite the use of medical cannabis, the majority of patients taking antidepressants and anxiolytic medications did not change their medications or the dose of these medications. Bouassa et al., found that THC and CBD were well-tolerated by patients with HIV on anti-retroviral therapy [9]. Their study did find that patients on high doses of CBD (800 mg/day) should be monitored for liver pathology.

Bacalia et al. examined the impact of CBD on intestinal inflammation, and they found that in female mice, CBD suppresses inflammation in the absence of estradiol, but it enhances inflammation in animals with estradiol [10]. Yekhtin and colleagues found that both THC and CBD reduced nitric oxide production by lipopolysaccharide (LPS)-stimulated peritoneal macrophages [11]. Interestingly, only CBD was able to reduce cytokine production in LPS-stimulated macrophages, suggesting differences in the anti-inflammatory properties of these two cannabinoids. Both cannabinoids were equally beneficial at improving clinical outcomes in the dextran sodium sulfate murine model of colitis.

Gáll and collaborators found that CBD increases the latency to the first seizure and decreased the mortality associated with the pentylenetetrazol (PTZ)-kindling model of epilepsy in rats [12]. However, no impact was observed for seizure frequency or duration in this model. In a placebo-controlled trial of cannabinoids (CBD and THC at a 20:1 ratio using both extracts and pure compounds), Schnapp et al. found no impact on sleep parameters in patients with autism spectrum disorder [13]. 

In another study that examined the anti-inflammatory activity of cannabinoids outside of the gastrointestinal tract, Lowin et al. found that the impact of THC on inflammation in synovial fibroblasts from rheumatoid arthritis (RA) patients is dose dependent [14]. Therefore, using THC to treat RA may require titrating the dose to find an effective dose for each patient. Golan and colleagues found that a novel compound (HU-585), a synthetic derivative of anandamide and oleic acid, induces apoptosis and senescence in treatment-resistant neuroblastoma cells [15]. Greiner et al. report that activation of the cannabinoid receptor 2 produces protective effects in vascular smooth muscle cells and cardiac myocytes, and the opposite effect is observed when cannabinoid receptor 1 is activated in these cells [16]. Finally, in a study by Mottarlini and colleagues, it was found that cannabidiol administered via intraperitoneal injection is able to be detected in the prefrontal cortex of the brain, and that CBD treatment modulates the expression of brain-derived neurotropic factor (BDNF) [17]. BDNF plays a role in neurodevelopment, neuroplasticity, and neuroprotection, and the authors propose that CBD may prove beneficial in these areas when taken as a supplement.

## 2. Review Articles, Brief Reports, and Systematic Reviews

Four review articles in this Special Issue summarize information on a wide array of topics related to cannabinoids. Duranti and collaborators review the current literature on the endocannabinoid system with regard to neuroprotection and neuroregeneration [18]. The focus of their review highlights the potential of modulating the endocannabinoid system to treat hypoxia–ischemia in newborns, which can lead to encephalopathy, a condition for which there are currently limited treatment options. Chacon et al. review some of the less abundant (“secondary”) terpenes found in *Cannabis* and the potential therapeutic utility of some of these molecules to treat a number of medical conditions [19]. Tudorancea and colleagues review the evidence that supports targeting the endocannabinoid system to treat age-related conditions such as Alzheimer’s disease, osteoarthritis, and hypertension [20]. Finally, Sionov and Steinberg examine the antimicrobial activity of endocannabinoids and phytocannabinoids. This latter review is important because the development of novel antibiotics is an ongoing need as resistance to current drugs is a continually evolving issue [21].

In a brief report by Sestan-Pesa et al. the authors explore the mechanism by which THC may lead to increased risk of schizophrenia, depression, and anxiety by looking at ghrelin signaling [22]. Ghrelin and its receptor, growth hormone secretagogue receptor (GHSR), have previously been found to play a role in anxiety- and depression-related behavior in animal models. However, the authors report no difference in anxiety-like behavior between wild-type and GHSR knockout animals after exposure to THC.

Licitra and colleagues performed a systematic review of the literature on cannabinoids in zebrafish [23]. The authors find that in many ways zebrafish respond similarly to rodents following cannabinoid exposure, and may serve as another useful model for studying the effects of cannabinoids in disease and on humans. 

## 3. Closing Remarks

*Cannabis* is a complicated plant that produces over 100 cannabinoids in addition to terpenes and flavonoids. Adding to the complexity of trying to address the mechanism of action for cannabis is the fact that the cannabinoids that have been studied have been reported to exhibit activity at a number of different receptors. This makes cannabinoids (and cannabis) a promiscuous drug. While typically viewed as a negative, promiscuous drugs do offer some advantages, most notably the ability to target different pathways of a disease with one medication [24]. The field of medical cannabis is growing rapidly, and as patients continue to use this plant to treat their conditions, there will remain a growing need for the scientific and medical communities to better understand how cannabis can impact the body. Not only is research needed to address the potential benefit and hazards of cannabis and individual cannabinoids, but also we must determine which routes of administration are best for each condition.
